# Deferral of elective surgeries during the COVID-19 pandemic and its impact on Palestinian patients: a cross-sectional study

**DOI:** 10.1186/s13031-023-00509-w

**Published:** 2023-03-24

**Authors:** Mousa Atary, Niveen M. E. Abu-Rmeileh

**Affiliations:** Institute of Community and Public Health, Birzeit University, Birzeit, Palestine

**Keywords:** Surgical deferral, Palestine, Anxiety and depression, Physical impact, Financial impact

## Abstract

**Background:**

During the Covid-19 epidemic, the increased number of people seeking medical attention worsened hospital shortages. This shortage required reallocating the workforce, personal protective equipment (PPE), medical equipment, medical disposables, and hospital wards. This reallocation delayed a number of elective surgeries. This study explored the financial, physical, and psychological implications of deferring elective surgeries on Palestinians in three West Bank hospitals during the pandemic.

**Methods:**

This cross-sectional study included 398 patients from tertiary hospitals in Palestine whose elective surgical procedures were deferred due to the COVID-19 pandemic. Between 8/8/2021 and 6/9/2021, data were collected on patients who had elective surgery deferral at three government hospitals in the West Bank of the Palestinian territories. There were five parts to the study tool; personal information, access to the health system, physical affection, financial effect, and psychological effect. Statistical analysis included a univariate, bivariate and multivariate.

**Results:**

The healthcare system's response to the COVID-19 epidemic directly affected patients whose surgeries were deferred. The healthcare system's response was the cause of the delay in 91.5% of the cases. Orthopedic and neurological surgeries account for 48.3% of deferred surgery. Other than delayed surgeries, 30.2% of patients were unable to get additional health care services. Physically, 55.5% of patients were impacted, 45% were anxious, and 29.6% were depressed.

**Conclusions:**

Patients who had procedures deferred as a result of the healthcare system's response to the COVID-19 epidemic were impacted physically, financially, and psychologically. There should bea better crisis management strategyto ensure that certain hospitals are able to operate regularly despite the situation.

## Background

**Fig. 1:**
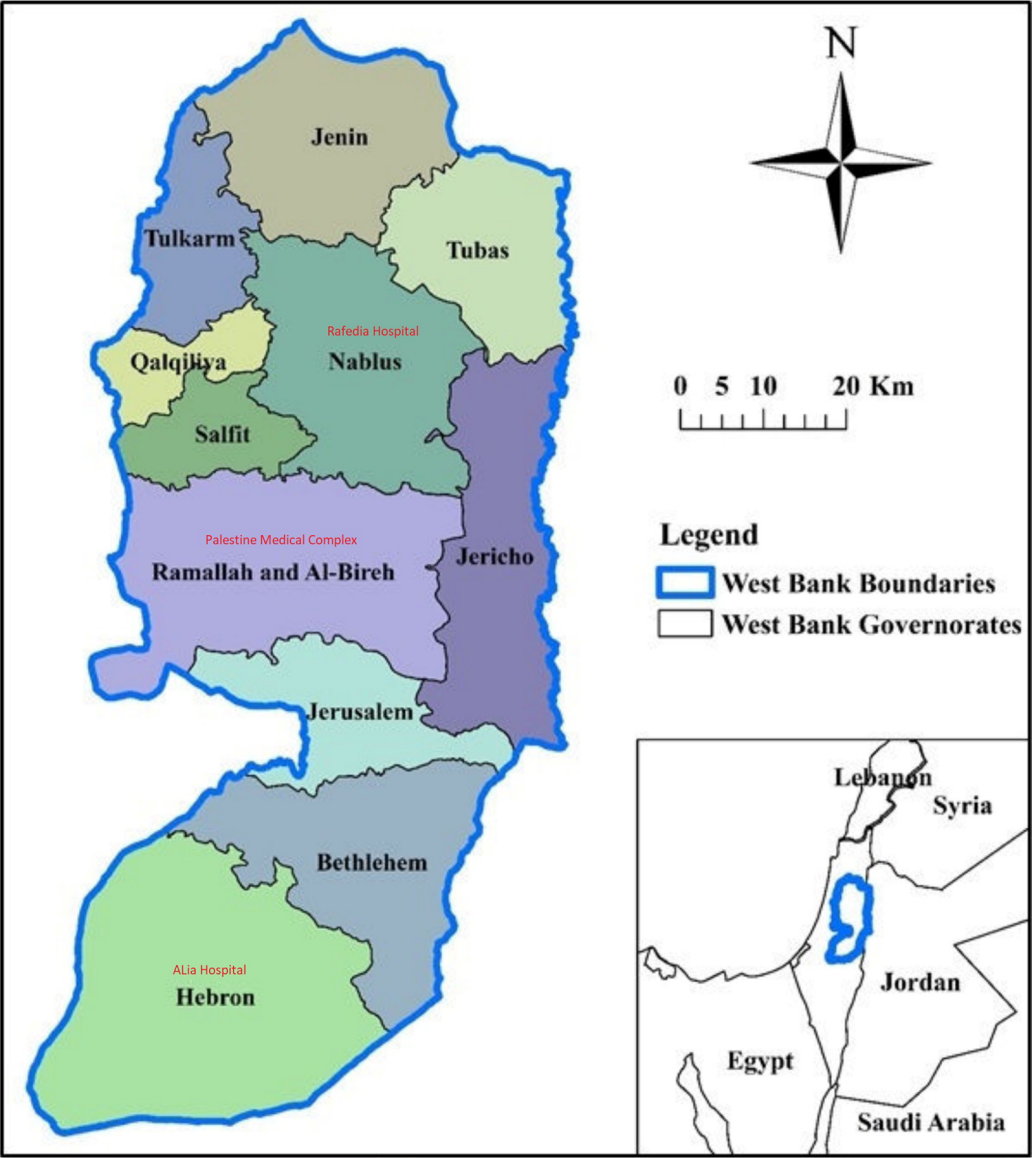
.

Most hospitals in developing countries lack a strong infrastructure, medical staff, and supplies, including protective gear and medications [1]. This scarcity could be quantifiable in some countries (where demand exceeds supply) or unevenly distributed among hospitals within the same country (the available resources are plenty but not dispersed fairly) [2]. In the majority of nations, COVID-19 patients exceeded hospital capacity. In addition, 17% of hospitalized COVID-19 patients required ventilators [4]. Sedatives and neuromuscular blockers were in high demand, and COVID-19 patients outnumbered ICU beds [4]. During the pandemic, personal protective equipment (PPE) was scarce due to high demand and China's export ban [6]. Departments moved resources to manage COVID-19 patients and satisfy demand [2–4]. To handle scarce resources, hospitals around the world shifted ventilators from operating rooms to intensive care units (ICUs). Move surgical teams to COVID-19 patients. Transformed other departments into COVID-19 inpatient departments and delayed surgeries [7–10]. Surgeries were delayed to minimize the amount of PPE needed to protect surgical teams and patients from infection, to free up surgical teams for COVID-19 ICU beds, and to prevent a shortage of medical staff by reducing their exposure to COVID-19. By delaying elective surgeries, surgeons could prioritize emergency surgeries [8, 9, 11, 12]. Delaying non-emergency procedures due to a shortage of PPE reduced medical staff's COVID-19 infection risk, thus preventing a shortage of medical professionals [10, 11, 13, 14]. In addition to the COVID-19 pandemic, the Palestinian health system struggled in the face of recurrent violence [15]. In 2002, the second intifada put a heavy strain on the Palestinian health system, including the physical separation of the West Bank and Gaza Strip and West Bank restrictions. Imports are slowed by actions taken by Israel, a lack of cash, and donations. So there is always a lack of medical equipment. Health teams got better at working in places with conflict and not *-22221112606563having enough resources [15, 16]. Most Palestinians live below the poverty line, forcing them to travel to government hospitals such as Palestine Medical Complex in Ramallah, Rafedia hospital in Nablus, and Alia hospital in Hebron [16]. According to Palestinian Ministry of Health records, 9396 elective surgeries were deferred in the three tertiary hospitals during the designated three two-week study period. This study examined the financial, physical, and psychological effects of COVID-19 related elective surgical deferral on Palestinians (Fig. 1).

## Methodology

### Study design

Between 8/8/2021 and 6/9/2021, data were collected for a cross-sectional study of patients who had elective operation deferral at three government hospitals in the West Bank of the Palestinian territories.

### Study population

The West Bank is divided into eleven governorates. Tulkarem, Jenin, Qalqelia, Nablus, Tubas, and Salfit comprise the governorates in the north. Ramallah and Al-Beireh, Jericho, and Jerusalem make up the central region. At the same time, Hebron and Bethlehem make up the southern district. The study's population included all Palestinian patients seeking treatment at three governmental hospitals in the West Bank. Rafedia hospital (located in the north of the West Bank, Nablus), Palestine Medical Complex (PMC) (the center, Ramallah and Al-Beireh), and Alia hospital (the south, Hebron) were included in the study. In a subsequent description, these hospitals will be referred to as the northern, central, and southern hospitals, respectively.

The sample frame of the analysis contained just the list of deferred elective procedures from the three hospitals during each phase of the COVID-19 pandemic (May/2020, September/2020, and January/2021). The operation list for these periods was collected from the Central Health Information System of the Palestinian Ministry of Health. According to these lists, 662 surgical interventions were deferred during the study period in the north, 724 in the center, and 587 in the south. With about 48 thousand elective surgeries deferred in the minister of health hospitals [2, 3], a 95% confidence interval, 5% error, and a variation of 50%, the sample size was determined to be 381 people using a sample size table. A participant was selected from these lists utilizing a systematic random sampling of every 5th patient on the list, and a phone call was made to that individual. First, the participants' verbal informed consent was obtained over the phone, and after that, the link to begin engaging in the survey was delivered over SMS. A second call was made to those who did not respond.

### Study tool

Using the KoBo Toolbox website, an online survey was conducted for the research. A questionnaire contained a different of validated tools. A pilot test was conducted on 14 patients prior to data collection.

The study tool is composed of five sections. The initial section contained personal information and data. The second section concerned accessing health services and the reasons for inaccessibility. The third section discussed the physical effects of postponing a procedure on the patient. The fourth section discussed the patient's financial impact ofpostponing the operation. The fifth section examined the psychological impact of postponing a procedure on the patient. The component of the questionnaire pertaining to personal information included age, gender, residency, history of chronic diseases, name of operations, the hospital where they were to be performed, history of COVID-19 infection, and whether this infection occurred at the same time as the surgery. The section of the questionnaire about the ability to receive health services used Arabic validated questionnaire of the Palestinian Central Bureau of Statistics (PCBS) titled Impact of COVID-19 on the Palestinian Households' Socio-Economic Conditions, 2020 [4]. This section contained eight primary questions. These included queries on the patient's need for health care other than the scheduled procedure. These health services included urgent and non-urgent surgeries, chronic and acute disease care, drug purchases, laboratory and radiological tests, and medical report or referral coverage. Each primary question had two sub-questions, the first regarding getting the medical treatment and the second explaining why it was inaccessible.

The section of the questionnaire about the physical impact of operation deferral on patients used the Arabic-validated version of the RAND 36-item health survey 1.0, which allowed for non-commercial use [5]. This part included 14 questions that cover the physical impact on upper limbs, lower limbs, ability to walk, and ability to work physically.

The section of the questionnaire about the financial impact of operation deferral on patients used Arabic validated Palestinian family survey, 2010 questionnaire by PCBS [6]. This part included seven questions that measure the work absence, its duration, its cause, the type of work before and after the COVID-19 pandemic, the cost of transportation for rescheduling the operation, and the cost of medications during the operation deferral period.

The section of the questionnaire about the psychological impact of operation deferral on patients used an Arabic-validated version of the hospital anxiety and depression scale (HADS) [7]. It consisted of fourteen questions, seven for measuring anxiety, and seven for measuring depression.

### Data manipulation

Age was categorized into four categories, 15 years each. The residence was categorized into north, center, and south. The individual operation name was transformed into the operation type, which was then grouped according to the department where the operation was performed. The subspecialty surgeries' category included (Ear, Nose, and Throat (ENT), maxillofacial, ophthalmology, vascular, and urology. The comorbidities were categorized into three categories (none, one, two or more). Finally, the number of health services that could not be accessed was categorized into five categories starting with no service and ending with four or more services.

The study had three primary outcomes: physical, psychological, and financial scores. The physical score was summed according to the RAND score and categorized into four categories (each quartile), then recategorized into two (affected or not affected). The sum of the score was also used as a continuous variable. The physical impact using the RAND score was utilized as a categorical for bivariate analysis and a continuous score for the regression. The financial score: Absence from work was categorized into four categories: no absence and absence lasting one, two, or three months or longer. The sum of the direct cost of transportation and medical was done then the results were categorized into three groups (mild, moderate, and severe impact). The psychological score: the sum of anxiety and depression scores from the HADS score was done (0–21) for each item used as a continuous variable, the recategorization in normal (0–7), borderline (8–10), and abnormal (11–21) categories were done according to the score instructions. The psychological impact was divided into two main categories: depression and anxiety. Both were measured using the HADS score, the categorical score used for bivariate analysis, and the continuous score used for regression.

All variables of interest were summarized using frequency and percent or mean and standard deviation (SD). Bivariate analyses were done using cross-tabulation and a one-way Anova test for all data. The multivariate analysis utilized Age and Sex adjusted regression for physical impact and all factor adjusted regression for psychological impact.

## Results

There were a total of 430 answered calls, seven patients were found to have passed away prior to the call and were therefore omitted from the study, and eight individuals declined to participate. SMS links were distributed to 415 individuals, and 402 completed questionnaires were submitted. On a second call to the remaining individuals, five individuals stated that their families encouraged them not to participate, and two individuals stated that they did not have enough time to participate. The remaining six individuals did not answer the second call. Four respondents indicated that their operations were urgent, so these were excluded from the calculations.

### Characteristics of study population “sociodemographic and clinical”

The study population was equally distributed according to gender. Patients from the center (35.4% (138/398)) and north (34.7%(141/398)) hospitals were equal and slightly more than cases from the south (29.9%(119/398)) hospitals. Patient's residence, according to the governorates, was (37.9%(151/398)) in the northern, (33.4%(133/398)) in the southern, and (28.6%(114/398)) in the central governorates. Patients aged 46–60 years were (36.2%(144/398)), followed by patients aged 31–45 years (28.4%(113/398)), and 20.9%(83/398) of the population was between 15 and 30 years old.. Most of the surgeries deferred were orthopedic surgeries (34.2%(136/398)), followed by neurosurgery surgeries (16.1%(64/398)). ENT surgeries in third place (12.1%(48/398)), then general surgery (10.6%(42/398)), gynecology surgeries (9.5%(38/398)), scope procedure (7.3%(29/398)). The least frequently deferred surgical interventions included urology surgeries (5.5%(22/398)), ophthalmology surgeries (2.5%(10/398)), vascular surgeries (1.3%(5/398)), and lastly, maxillofacial surgeries (1%(4/398)). A majority of the patients had no comorbidities (63.6%(253/398)), and only 18.1%(72/398) had one comorbidity. 56.5%(225/398) of patients declared they were not infected with COVID-19 before, 34.9%(139/398) were infected not during the scheduled surgery time, and 8.5%(34/398) were infected at the operation time. Aside from elective surgery, 69.8% (278/398) of patients reported having access to all necessary health care. The remaining 20% (83/398) were unable to access at least one additional health service, 6.5% (26/398) were unable to access two additional services, 2.5% (10/398) were unable to access three additional services, and 0.3%(1/398) were unable to access four or more services. During the COVID-19 pandemic, 42.5%(169/398) of patients continued to work, 28.4%(113/398) did not work for three months or more, and 14.6%(58/398) did not go to work for one or two months (47.2%(108/398)). owing to COVID-19 infection or quarantine, (26.2%(60/398)) due to other diseases (including the disease for which they had scheduled surgery), and (26.6%(61/398)) due to administrative orders. Surgery deferral costed more than 200 new Israeli Shekel (about 65 united states dollars) for transportation in 33.7%(134/398) of patients and for medication in 15.4%(61/398). An abnormal anxiety scale was found in 45%(179/398) of patients, while an abnormal depression scale was found in 29.6%(118/398). There was no physical affection in 44.5%(177/398) of patients, 28.4(113/398) with minimal affection, 21.9%(87/398) with moderate, and 5.3%(21/398) with severe physical affection (Table 1).

**Table 1:** Characteristics of the study population

Variable	Items	Frequency (N = 398)	Percentage
Age groups	15–30y	83	20.9
	31–45y	113	28.4
	46–60y	144	36.2
	> 60y	58	14.6
Gender	Female	202	50.8
	Male	196	49.2
Hospital*	Centre Hospital	138	34.7
	North Hospital	141	35.4
	South Hospital	119	29.9
Residence in West Bank**	North	151	37.9
	Centre	114	28.6
	South	133	33.4
Type of surgery deferred	Ear, Nose, and Throat	48	12.1
	Gynecology	38	9.5
	Maxillofacial surgery	4	1.0
	Neuro	64	16.1
	Ophthalmology	10	2.5
	Orthopaedic	136	34.2
	Scopes	29	7.3
	General Surgery	42	10.6
	Urology	22	5.5
	Vascular surgery	5	1.3
No. of comorbidities	No comorbidities	253	63.6
	One comorbidity	72	18.1
	Two comorbidities	36	9.0
	Three comorbidities	26	6.5
	Four comorbidities	10	2.5
	Five comorbidities	1	0.3
Covid-19 infection	Yes, at the time of deferral of surgical intervention	34	8.5
	Yes, other than the time of deferral of surgical intervention	139	34.9
	No	225	56.5
No. of health services that could not be accessed by the deferred operation patients***	Accessed all other health services	278	69.8
	One health services	83	20.9
	Two health services	26	6.5
	Three health services	10	2.5
	Four health services	1	0.3
Duration of absence from work during the covid-19 pandemic	No	169	42.5
	1-month	58	14.6
	2-months	58	14.6
	Three months or more	113	28.4
Causes of absence from work	Administrative orders	61	26.6
	Covid-19 infection or quarantine	108	47.2
	Disease (includes the diseases for which surgery was scheduled)	60	26.2
Cost of transportation due to surgery deferral	Less than 100 NIS	126	31.7
	100–200 NIS	138	34.6
	200–300 NIS	78	19.6
	More than 300 NIS	56	14.1
Cost of medications due to surgery deferral	Less than 100 NIS	268	67.3
	100–200 NIS	69	17.3
	200–300 NIS	19	4.8
	More than 300 NIS	42	10.6
Anxiety scale (HAAD)	Normal	106	26.6
	Borderline	113	28.4
	Abnormal	179	45.0
Depression scale (HAAD)	Normal	126	31.7
	Borderline	154	38.7
	Abnormal	118	29.6
Physical affection of patient due to surgical deferral	No Effect	177	44.5
	Minimal Effect	113	28.4
	Moderate Effect	87	21.9
	Severe Effect	21	5.3

Characteristics of the study population, Palestine, 2020–2021

*Centre hospital is Palestine Medical Complex, the North hospital is Rafedia hospital, and the south hospital is Alia hospital

**Nothern governorates include (Jenin, Qalqelia, Tubas, Salfet, Nablus, and Tulkarem), Centre governorates include (Jerusalem, Ramallah, and Jericho), and south governorates include (Hebron and Bethlehem)

***Health services include urgent and non-urgent surgeries, chronic and acute disease care, drug purchases, laboratory and radiological tests, and medical report or referral coverage

### The financial impact

The financial impact was estimated using two variables: the length of absence from work and the direct cost of postponing surgery. Transportation and pharmaceutical costs were used to compute the direct cost, which was then categorized as having a mild (28.4%), moderate (55.5%), or severe (16.5%) impact. The direct cost was directly proportional to the patient's age; the older the patient, the greater the financial impact (77.6% versus 28.9%) (P-value 0.001). The direct cost was highly related to the procedure type (P-Value 0.001). The financial impact was greater in patients undergoing neurosurgery (severe impact in 28.1%, moderate impact in 68.8%) and orthopedic surgery (severe impact in 24.3, moderate impact in 48.5%) than in patients undergoing other surgeries. The severity of the financial impact grew as the patient's comorbidities increased (p-value = 0.001). With two or more comorbidities, 23.3% had a severe impact, 19.4% had one comorbidity, and 13% had none. In addition, the severity of the financial impact increased as the number of inaccessible health services increased (P-value = 0.009) (severe impact in 100% of patients with four inaccessible health services). Lastly, the physical impact of surgical postponement was associated with a greater financial burden on the patient (P-value 0.001) (Table 2).

**Table 2:** Financial impact on patients with deferred surgery

Variable	Item	The direct cost impact on participants (cost of transportation and medications due to surgical deferral)*			Pearson Chi-square	Pearson Chi-square P-value ≤ 0.05 is statistically significant
		Mild (%)	Moderate (%)	Severe (%)		
Age groups	15–30y	50.6	42.2	7.2	42.397	.000
	31–45y	31.9	59.3	8.8		
	46–60y	17.4	59.7	22.9		
	> 60y	17.2	56.9	25.9		
Gender	Female	29.7	54.5	15.8	.348^a^	.840
	Male	27.0	56.6	16.3		
Hospital of scheduled operation	Centre Hospital	26.1	52.9	21.0	9.159	.057
	North Hospital	25.5	56.7	17.7		
	South Hospital	34.5	57.1	8.4		
Residence in West Bank	North	23.8	55.6	20.5	5.517	.238
	Centre	30.7	57.9	11.4		
	South	31.6	53.4	15.0		
Type of operation	Subspeciality**	38.2	53.9	7.9	47.948^a^	.000
	Gynecology	39.5	50.0	10.5		
	Neurosurgery	3.1	68.8	28.1		
	Orthopaedic	27.2	48.5	24.3		
	General Surgery	35.2	62.0	2.8		
No. of comorbidities	None	35.6	51.4	13.0	19.258	.001
	One	15.3	65.3	19.4		
	Two or more	16.4	60.3	23.3		
Covid-19 infection regarding the time of scheduled operation	Did not infected	22.4	59.2	18.4	4.686	.321
	Infected at other time than surgery time	29.7	55.7	14.7		
	Infected during surgery time	37.0	40.7	22.2		
The number of health services other than elective surgery which need by the participants	No services needed	31.3	56.1	12.6	20.255	.009
	One service	24.1	56.6	19.3		
	Two services	11.5	50.0	38.5		
	Three services	30.0	50.0	20.0		
	Four services	0	0	100.0		
Absence of work during the pandemic	Go to work all the time	34.9	50.9	14.2	21.480	.002
	1-month absence	31.0	60.3	8.6		
	2-months absence	29.3	62.1	8.6		
	Three or more months of absence	16.8	56.6	26.5		
The physical impact of surgical deferral	Not affected physically	40.7	53.7	5.6	38.712	.000
	Affected physically	18.6	57.0	24.4		

Univariate analysis of the financial impact on patients with deferred surgery, Palestine, 2020–2021

^a^0 cells (0.0%) have expected count < 5

*Mild impact is < 200 Israeli Shekels, Moderate imapct is 200-500Israeli Shekel, Severe impact is > 500 Israeli Shekels

**Subspeciality surgeries include ENT, Ophthalmology, vascular, and maxillofacial surgeries

### The physical impact

The physical impact of deferring a surgical intervention increased significantly with age (77.6% for those older than 60, 70.1% for those aged 46–60, 45.1% for those aged 30–45, and 28.8% for those under 15–29). It was significantly associated with operation type (P-value 0.001). Physical impact was experienced by 95.3% of neurosurgery patients and 75% of orthopedic surgery patients. Comparatively, fewer gynecological patients (47.4%), general surgery patients (26.9%), and subspecialty surgery patients (ENT, Ophthalmology, vascular, and maxillofacial surgeries) (23.6%) were affected. The patient's physical effect grew as the number of comorbidities increased. Seventy-seven percent of physical impact was related to two or more comorbidities, 66.7% with one, and 46.2% with none. The greater the patient's physical impact, the more health services they were unable to access (100% with the inability to access four services, 90% with three services, 80.8% with two services, 62.7% with one service, 49.6% with no services). The increasing direct cost of surgical deferral was significantly related to physical impact (84.4% in severe financial impact, 57% in moderate impact, and 36.3% in mild impact). Significant correlations between physical impact and anxiety and depression were found to be above 64% in abnormal people and under 39.7% in normal people.

Age and Sex adjusted linear regression showed that physical impact is statistically significantly related to age (P-value = 0.001), the type of operation (Neurosurgery and orthopedic both with P-value < 0.001), the number of health services other than surgery that the patient could not access (two services with P-value = 0.01, three services with P-value = 0.005) (Table 3).

**Table 3:** Physical impact on patients with deferred surgeries

Variable	Item	The physical impact of deferring surgery (%)		Pearson Chi-square (%)	Pearson Chi-Square P-value ≤ 0.05 is statistically significant	Mean score	Mean score p-value ≤ 0.05 is statistically significant	Coefficient	Coefficient p-value ≤ 0.05 is statistically significant
		No effect	Affected						
Age groups	15–30y	71.1	28.9	52.625	.000	1.2892	.000	.123	.001
	31–45y	54.9	45.1			1.4513			
	46–60y	29.9	70.1			1.7014			
	> 60y	22.4	77.6			1.7759			
Gender	Female	48.0	52.0	2.090	.148	1.5198	.149		
	Male	40.8	59.2			1.5918			
Hospital	Centre Hospital	38.4	61.6	5.596^a^	.061	1.6159	.061		
	North Hospital	43.3	56.7			1.5674			
	South Hospital	52.9	47.1			1.4706			
Residence in West Bank	North	40.4	59.6	1.655	.437	1.5960	.439		
	Centre	46.5	53.5			1.5351			
	South	47.4	52.6			1.5263			
Type of operation	Subspeciality surgery*	76.4	23.6	123.471	.000	1.2360	.000	.155	.900
	Gynecology	52.6	47.4			1.4737		2.817	.066
	Neurosurgery	4.7	95.3			1.9531		12.245	.000
	Orthopedic	25.0	75.0			1.7500		9.506	.000
	General Surgeries	73.2	26.8			1.2676			
No. of comorbidities	None	53.8	46.2	25.712	.000	1.4625	.000	0.084	.934
	One	33.3	66.7			1.6667			
	Two or more	23.3	76.7			1.7671			
Covid-19 infection regarding the time of scheduled operation	Did not infected	37.8	62.2	2.402	.301	1.6224	.303		
	Infected at other time than surgery time	46.5	53.5			1.5185			
	Infected during surgery time	48.1	51.9			1.5348			
The number of health services other than elective surgery which need by the participants	No services needed	50.4	49.6	17.929	.001	1.4964	.001		
	One service	37.3	62.7			1.6265		1.291	.180
	Two services	19.2	80.8			1.8077		4.031	0.01
	Three services	10.0	90.0			1.9000		6.673	.005
	Four services	0.0	100.0			2.0000			
Absence of work during the Pandemic	Go to work all the time	47.9	52.1	5.405	.144	1.5207	.145		
	1-month absence	46.6	53.4			1.5345			
	2-months absence	50.0	50.0			1.5000			
	Three or more months of absence	35.4	64.6			1.6460			
The direct Cost impact of surgical deferral	Mild impact	63.7	36.3	38.712	.000	1.3628	.000		
	Moderate impact	43.0	57.0			1.5701			
	Severe impact	15.6	84.4			1.8438			
Anxiety Level (HAAD score)	Normal	64.2	35.8	23.121	.000	1.3585	.000		
	Borderline	39.8	60.2			1.6018			
	Abnormal	35.8	64.2			1.6425			
Depression level (HAAD score)	Normal	60.3	39.7	19.225	.000	1.3968	.000		
	Borderline	39.0	61.0			1.6104			
	Abnormal	34.7	65.3			1.6525			

Univariate and multivariate analysis of the physical impact on patients with deferred surgeries, Palestine, 2020–2021

*Subspeciality surgeries include ENT, Ophthalmology, vascular, and maxillofacial surgeries

### The psychological impact

Depression was significantly related to age groups (P-value < 0.001); the depression score increased with age, reaching the maximum of abnormality in the 45–60 years age group (39.6%), while it decreased to (34.5%) in the above 60 years age group. In addition, it was significantly related to the type of operation (P-value = 0.001); the depression scores were (6.3% normal and 42.2% abnormal) in neurosurgery patients, (28.9% normal, and 31.6% abnormal) in gynecology patients, (34.6% normal and 27.9% abnormal) in orthopedic patients, (35.2% normal and 23.9% abnormal) in general surgery patients, and (43.8% normal and 27% abnormal) in subspeciality surgery patients. Depression increased with the COVID-19 infection (P-value = 0.023); Abnormal HADS score was found in 26.5% of whom did not infect with COVID-19 before, 28.6% of whom got infected with COVID-19 at other times than surgery time, and 51.9% of whom infected with COVID-19, during surgery time.

Depression score was significantly positively related to the number of health services the patients could not access. For example, patients requiring four services other than elective surgery were 100 percent depressed, compared to those requiring three (60%) and two (50%) services. In comparison, one service (30.1%) and no necessary services (26.3%) were required.

Depression related to job absence duration (44.2% for three months or more, 32.8% for two months, and 12.2% for one month). Moreover, it was related to the direct cost impact of surgical postponement; 46.9% for severe impact and 26.5% for light impact. It was additionally related to the physical effects of surgical postponement; 34.8% were physically affected whereas 23.2% were not.All parameters adjusted linear regression of depression was done. Depression score was statistically significantly related to age (P-value < 0.001), the number of health services the patient could not access was three or more (P-value = 0.036), and the absence of work duration was three months or more (P-value = 0.001) (Table 4).

**Table 4:** Depression scores of the patients with deferred surgeries

Variable	Item	Depression status (HADS score)*			Pearson Chi-square	Pearson Chi-square P-value ≤ 0.05 is statistically significant	Mean score	Mean score p-value ≤ 0.05 is statistically significant	Coefficient	Coefficient p-value ≤ 0.05 is statistically significant
		Normal (%)	Borderline (%)	Abnormal (%)						
Age groups	15–30y	54.2	31.3	14.5	36.870	.000	6.6988	.000	9.482	< 0.001
	31–45y	35.4	38.9	25.7			8.9292			
	46–60y	18.1	42.4	39.6			9.7917			
	> 60y	25.9	39.7	34.5			10.0172			
Gender	Female	34.7	36.1	29.2	1.881	.390	8.6881	.265		
	Male	28.6	41.3	30.1			9.1888			
Hospital	Centre Hospital	31.9	39.9	28.3	1.294	.862	9.1667	.753		
	North Hospital	31.9	35.5	32.6			8.8227			
	South Hospital	31.1	41.2	27.7			8.7983			
Residence in West Bank	North	31.1	35.8	33.1	2.318	.677	9.0861	.854		
	Centre	34.2	37.7	28.1			8.9035			
	South	30.1	42.9	27.1			8.7895			
Type of operation	Subspeciality surgery**	43.8	29.2	27.0	27.460	.001	8.2697	.000		
	Gynecology	28.9	39.5	31.6			9.3684			
	Neurosurgery	6.3	51.6	42.2			11.1875			
	Orthopedic	34.6	37.5	27.9			8.5221			
	General Surgeries	35.2	40.8	23.9			8.2958			
No. of comorbidities	None	35.6	39.1	25.3	8.282	.082	8.4348	.007	0.648	.517
	One	26.4	38.9	34.7			9.4028			
	Two or more	23.3	37.0	39.7			10.2055			
Covid-19 infection regarding the time of scheduled operation	Did not infected	30.6	42.9	26.5	11.302	.023	9.0306	.000		
	Infected at other time than surgery time	34.4	37.0	28.6			12.3333			
	Infected during surgery time	7.4	40.7	51.9			8.5641			
The number of health services other than elective surgery which need by the participants	No services needed	34.5	39.2	26.3	16.400	.037	8.4209	.000	0.110	0.913
	One service	31.3	38.6	30.1			9.3976			
	Two services	15.4	34.6	50.0			11.4615		1.475	0.141
	Three services	0.0	40.0	60.0			12.5000		2.107	0.036
	Four services	0.0	0.0	100.0			12.0000			
Absence of work during the pandemic	go to work all the time	40.2	34.9	24.9	38.501	.000	8.0473	.000		
	1-month absence	48.3	39.7	12.1			7.3103			
	2-months absence	25.9	41.4	32.8			9.2069			
	Three or more months of absence	13.3	42.5	44.2			10.9558		3.318	0.001
The direct Cost impact of surgical deferral	Mild impact	43.4	30.1	26.5	24.225	.000	7.8496	.000		
	Moderate impact	31.7	42.1	26.2			8.8688			
	Severe impact	10.9	42.2	46.9			11.0781			
The physical impact of surgical deferral	not affected physically	42.9	33.9	23.2	19.225	.000	7.8249	.000	1.269	0.205
	affected physically	22.6	42.5	34.8			9.8235			

Univariate and multivariate analysis of the depression scores of the patients with deferred surgeries, Palestine, 2020–2021

*The Hospital Anxiety and Depression Scale

**Subspeciality surgeries include ENT, Ophthalmology, vascular, and maxillofacial surgeries

Anxiety scores showed nearly similar relationships to depression but with increased abnormality scores. Age was significantly related to anxiety (P-value < 0.001), with 55.6% abnormalities in the 45–60 years age group and 46.2% abnormalities in the over 60 years age group. Type of operation was significantly related to anxiety (P-value < 0.001); HADS scores for anxiety were (1.6% normal, 68.6% abnormal) in neurosurgery patients, (31% normal, 46.5% abnormal) in general surgery patients, (30.9% normal, and 40.4% abnormal) in orthopedic patients, (32.6 normal and 41.6% abnormal) in subspeciality surgeries patients, and (31.6% normal and 26.3% abnormal) in gynecology patients). COVID-19 infection was significantly related to anxiety (P-value < 0.001). The abnormal anxiety score was 46.9% of patients did not infect with COVID-19, 40.3% of patients infected with COVID-19 other than the surgery, and 85.2% of patients infected with COVID-19 during surgery. The anxiety score was significantly positively related to the number of health services that could not be accessed by the patients other than the surgery (56.2% with two or more services, 47.2% in one service, and 41.1% with no services), the absence of work duration (62.8% in 3 or more months, 58.6% in two months, and 25.9% in one month), and the physical impact of surgery deferral (52%in physically affected and 36.2% in not affected).

All parameters adjusted linear regression of anxiety showed that anxiety score was statistically significantly related to age (p-value < 0.001). The patient was infected with COVID-19 during the planned operation time (P-value < 0.001). The patient was absent from work for two months or more (P-value < 0.001) (Table 5).

**Table 5:** Anxiety score of patients with deferred surgeries

Variable	Item	Anxiety status (HAAD score)*			Pearson Chi-square	Pearson Chi-square P-value ≤ 0.05 is statistically significant	Mean score	Mean score p-value ≤ 0.05 is statistically significant	Coefficient	Coefficient p-value ≤ 0.05 is statistically significant
		Normal (%)	Borderline (%)	Abnormal (%)						
Age groups	15–30y	48.2	25.3	26.5	30.738	.000	7.6	< 0.001	3.839	< 0.001
	31–45y	25.7	30.1	44.2			9.7522			
	46–60y	17.4	27.1	55.6			10.7361			
	> 60y	20.7	32.8	46.6			10.8103			
Gender	Female	30.2	27.2	42.6	2.679^a^	.262	9.7030	.672		
	Male	23.0	29.6	47.4			9.9337			
Hospital	Centre Hospital	23.9	27.5	48.6	1.377	.848	10.1087	.641		
	North Hospital	27.7	29.8	42.6			9.5816			
	South Hospital	28.6	27.7	43.7			9.7563			
Residence in West Bank	North	26.5	29.1	44.4	.434	.980	9.7483	.887		
	Centre	25.4	27.2	47.4			10.0000			
	South	27.8	28.6	43.6			9.7368			
Type of operation	Subspeciality surgery**	32.6	25.8	41.6	33.555	.000	9.4944	.000		
	Gynecology	31.6	42.1	26.3			9.3947			
	Neurosurgery	1.6	29.7	68.8			12.3281			
	Orthopedic	30.9	28.7	40.4			9.1912			
	General Surgeries	31.0	22.5	46.5			9.3803			
No. of comorbidities	None	30.8	28.1	41.1	9.383	.052	9.2095	.001		
	One	25.0	27.8	47.2			10.2500			
	Two or more	13.7	30.1	56.2			11.4932			
Covid-19 infection regarding the time of scheduled operation	Did not infected	20.4	32.7	46.9	23.425	.000	10.0918	.000		
	Infected at other time than surgery time	31.1	28.6	40.3			14.2963		1.173	0.241
	Infected during surgery time	3.7	11.1	85.2			9.2747		3.387	0.001
The number of health services other than elective surgery which need by the participants	No services needed	30.2	29.9	39.9	15.959	.043	9.2518	.000		
	One service	21.7	27.7	50.6			10.4458			
	Two services	15.4	15.4	69.2			12.0000		1.668	0.096
	Three services	0.0	30.0	70.0			14.4000		1.305	0.193
	Four services	0.0	0.0	100.0			12.0000		1.794	0.074
Absence of work during the pandemic	go to work all the time	38.5	26.6	34.9	46.369	.000	8.7041	.000		
	1-month absence	34.5	39.7	25.9			8.1897		4.163	< 0.001
	2-months absence	17.2	24.1	58.6			11.1897		4.726	< 0.001
	Three or more months of absence	9.7	27.4	62.8			11.6106		3.882	< 0.001
The direct Cost impact of surgical deferred	Mild	23.0	39.8	37.2	26.812	.000	8.8673	.000		
	Moderate	32.1	40.7	27.1			9.7059			
	Severe	25.0	68.8	6.3			11.8750			
The physical impact of surgical deferral	Not affected physically	38.4	25.4	36.2	23.121	.000	8.7288	.000		
	Affected physically	17.2	30.8	52.0			10.6878		1.332	0.184

Univariate and multivariate analysis of anxiety score of patients with deferred surgeries, Palestine, 2020–2021

^a^0 cells (0.0%) have expected count < 5

*The Hospital Anxiety and Depression Scale

**Subspeciality surgeries include ENT, Ophthalmology, vascular, and maxillofacial surgeries

## Discussion

The impact of COVID-19 on patients was not lim to physical aspects. Our study indicated that patients with deferred operations were affected physically, psychologically, and financially. In addition, due to poverty, 52% of Palestinian families were required to use government hospitals for health care [8]. The shutdown of these government hospitals during the crisis left people with little option but to wait and endure the consequences of delay [8]. Therefore, the government health sector should increase its efforts and resources to deal with the new crisis in order to mitigate its negative impact on patients.

The number of health services that patients could not obtain may indicate the severity of their sickness. Typically, more severely affected patients might seek out any means possible to undergo surgery [9]. They might request a report or referral to end their suffering, or they could repeat the laboratory and radiological tests to prove to the physician that they required immediate case treatment [9]. On the other hand, the more services a patient requires, the more severe the illnesses might eventually become [10]. Significant correlation existed between the number of inaccessible health services and the severity of the physical disability. This relationship's explanation was unclear, but its endurance should prompt more investigation [10].

The direct physical effect of surgical deferral during the COVID-19 pandemic was intimately linked to orthopedic and neurosurgical procedures [11]. If this type of surgery was not performed, it could damage the patients' mobility [11]. Due to the deferral of orthopedic procedures involving the lower extremities, particularly knee and hip arthritis, patients' mobility could be significantly impaired [11]. Other orthopedic procedures, such as operations on the upper limb, might alter patients' function but did not affect their mobility. Overall, the combination of persistent discomfort and limited joint range of motion would impair the patient's ability to do various physical tasks at home or at work. A study conducted in the United Kingdom revealed that at least 65 percent of patients would have an improvement in their physical and mechanical health after elective orthopedic surgery, particularly knee surgery [11].

The majority of elective neurosurgical procedures included spine operations (both lumbar and cervical spine). It was anticipated that postponing these operations would prolong the severity of pain, paresthesia, or muscle weakening in one or more limbs [12]. Therefore, the postponement would damage the patients' mobility and might impair their physical hand function in daily or occupational responsibilities [12]. The physical condition of patients undergoing spine surgery who had a disability of 40.1% before surgery improved at an atypical rate of 2.1 to one [12]. Therefore, delaying these operations would maintain these individuals with this handicap, if not worsening with prolonged durations of no treatment.

Age was related to the physical deferral of operations in numerous ways. Delaying the surgeries of elderly patients (who typically suffer from back or joint discomfort) would have a greater impact on them than on younger patients, who typically had different procedures, such as lipoma, ingrowing toenails, or peripheral nerve release [10]. As their muscle strength diminished, older people with musculoskeletal illnesses could not manage the physical restrictions imposed by these conditions [10]. In addition, a study revealed that older adults typically experienced poor sleep quality, which worsened their physical disability regardless of their psychological health [10]. They tended to assume that physical incapacity caused by their diseases was normal and impacted them far more than younger patients with the same condition [10].

The psychological effect of the surgical deferral appeared more clearly in anxiety than depression. Anxiety was intended to be viewed as a short-term psychiatric condition, but depression was believed to have a longer duration. Several studies demonstrated that anxiety symptoms were more severe than depression symptoms among individuals [13, 14].

The absence of work during the pandemic could trigger anxiety status easier than depression [15]. The absence of two months or more was enough to raise anxiety, while at least three months were related to depression. The financial impact was an intermediate factor for the indirect psychological impacts of surgical deferral. These findings were supported by the results of deferring total knee surgery due to the COVID-19 pandemic [15].

Depression was closely related to the inability to access health services. Frustration from this inability would trigger depression and increase the feeling of disability [15]. The need for health services on its own was considered a shortage and could have a psychological impact on the patient. Therefore, the inability to access them could be considered a double-bladed sword, triggering depression [15].

Patients' fear of COVID-19 itself. The dread of infecting family members, the concept of deferring the procedure and the potential harm or discomfort the patient might endure until his rescheduled appointment, the fear of deferring the surgery, the fear of hospitalization due to COVID-19 infection, or the fear of death [16, 17]. All factors resulted in increased anxiety levels. These interrelated reasons were consistent with findings from studies on the mental health of people during the COVID-19 epidemic [16, 17]. The severity of the psychological impact varied according to context, particularly understanding of the disease and its transmission method. However, other factors, such as quarantine, the inevitable deferral of management, and the financial burden of the crisis, all contributed to an increase in anxiety and depression [16, 17].

Those between the ages of 45 and 60 had the highest rates of abnormal anxiety and depression since this was the working age group; members of this age range typically had significant financial commitments, including the expense of children's education and family obligations [18]. As a result, delaying surgery within this age group would have a greater impact than among the elderly (those 60 and up), who typically had less financial responsibility and more religious faith [18]. Studies found that in the COVID-19 pandemic, there were two types of psychological impact; fear of the infection and fear of disease complications in the end. These were more prominent in the younger people than the adult [19, 20].

The planning of crisis management on a global scale, especially by the World Health Organization, should account for crisis collateral harm. For instance, in the COVID-19 outbreak, the non-COVID-19 patient's health should be considered from the outset in order to minimize the negative influence on their health. The initial phase of a crisis was always the most difficult, but when things begin to stabilize, there should be a division between the services supplied to maintain health services in a near-normal manner and the crisis response [21]. Separation was difficult during a crisis; therefore, preparation is the ideal method for avoiding the harmful effects of the crisis.

Palestine had been in a constant state of war and emergency. The health care system had developed a strategy for adapting to emergency conditions and their implications. During the COVID-19 pandemic, the crisis management committee handled situation management. However, our research indicated that extra attention should be paid to the members and specialties of the crisis management committee. This committee should consist of a multidisciplinary group, including psychologists, in order to minimize the psychological impact of the decisions made during the crisis. The empirical decision, which included cessation of living features such as elective health treatments, should be taken on a minimally required basis to minimize the psychological impact on individuals from being maximized.

### Limitation of the study

The questionnaire was built upon validated Arabic tools. The psychological tools were not built around the Palestinian context. However, it is an Arabic version validated and tested and found useful. We used an online tool, which provided an easy and cost-effective way to collect data in the pandemic era, with fewer errors while entering data on a computer compared to using paper, and it gave the ability to perform an extensive study at country or international levels. Still, an online survey had no interviewers, limiting the ability to clarify questions for each person per need [22]. The tool covered people with deferred elective operations; we had limitations in assessing emergency 'patients' operations deferral. We are limited in knowing the duration of deferral, the times of deferral, and the result of whether the patient could undergo the operation later. Another limitation was the psychological tool's inability to determine whether the outcomes were attributable to the pandemic or the deferral of surgery.

## Conclusion

The healthcare system's response to the COVID-19 epidemic directly affected patients whose surgeries were deferred. The healthcare system's response was the cause of the delay in 91.5% of the cases.. Orthopedic and neurological surgeries account for 48.3% of deferred surgery. Other than delayed surgeries, 30.2% of patients were unable to get additional health care services. Physically, 55.5% of patients were impacted, 45% were anxious, and 29.6% were depressed. However, the health system should have a better crisis management strategy that considers all of the services offered, construct or designate specialist hospitals for such conditions, and comprise crisis management committee members with experts from various disciplines.

## Data Availability

Tables were included in the manuscript; raw data is available with the author.
